# Green synthesis of *Nerium oleander*-conjugated gold nanoparticles and study of its in vitro anticancer activity on MCF-7 cell lines and catalytic activity

**DOI:** 10.1186/s40580-018-0142-5

**Published:** 2018-04-19

**Authors:** Abir Chandan Barai, Koushik Paul, Aditi Dey, Subhankar Manna, Somenath Roy, Braja Gopal Bag, Chiradeep Mukhopadhyay

**Affiliations:** 1 Department of Chemistry and Chemical Technology, Vidyasagar University, Midnapore, West Bengal 721102 India; 2 Department of Human Physiology with Community Health, Vidyasagar University, Midnapore, West Bengal 721102 India

**Keywords:** *Nerium oleander*, Green synthesis, Gold nanoparticle, Catalyst, Anti-cancer

## Abstract

The phytochemicals present in the stem bark extract of *Nerium oleander* (commonly known as Karabi) have been utilized for the green synthesis of stable gold-conjugated nanoparticles at room temperature under very mild conditions. The green synthesized gold-conjugated nanoparticles were characterized by surface plasmon resonance spectroscopy, High resolution transmission electron microscopy, X-ray diffraction studies and dynamic light scattering. A mechanism for the synthesis and stabilization of gold-conjugated nanoparticles (AuNPs) has been proposed. Anticancer activity of the stabilized AuNPs studied against MCF-7 breast cancer cell line revealed that the stabilized AuNPs were highly effective for the apoptosis of cancer cells selectively. The antioxidant activity of the stem bark extract of *Nerium oleander* has also been studied against a long lived 2,2-diphenylpicrylhydrazyl radical at room temperature. Moreover, the utilization of the stabilized AuNPs as a catalyst has also been demonstrated.

**Electronic supplementary material:**

The online version of this article (10.1186/s40580-018-0142-5) contains supplementary material, which is available to authorized users.

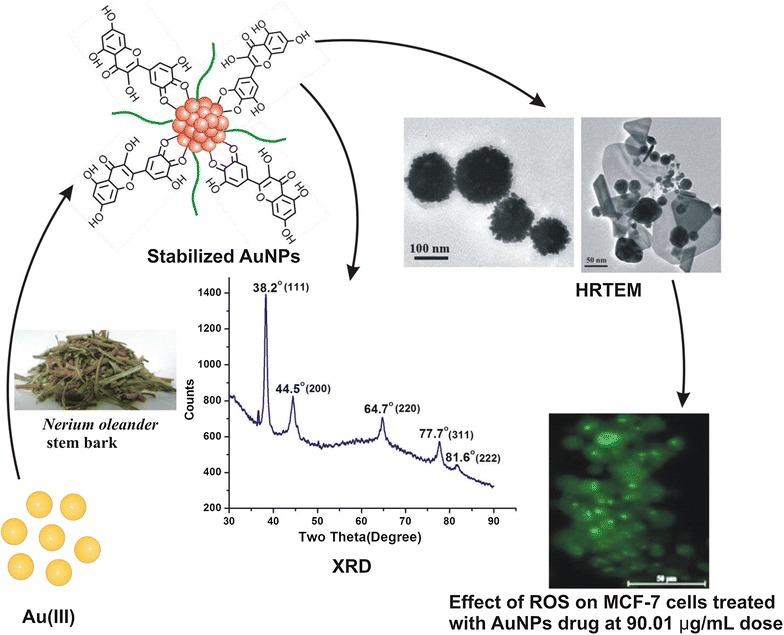

## Introduction

Metal nanoparticles has been area of intense research interest in recent years because of their applications in diversified areas such as catalysis, cancer therapy, drug delivery, medicine, biotechnology, electronics, etc. [1–7]. Among various metal nanoparticles, the studies on gold nanoparticles (AuNPs) have been carried out most extensively due to its least toxicity to animal and microorganism cells compared to the other metal nanoparticles. AuNPs have different physicochemical properties compared to the bulk solids because of its large surface to volume ratio. Depending upon their shape, size, degree of aggregation and stabilizing ligands, AuNPs exhibit different colors [8–11]. AuNPs are widely applied in enzyme immobilization in biosensor for the detection of virus, bacteria and pathogen and in biomedical science such as the nanobiodiagnostics and controlled drug delivery [12–14]. It has also been applied in surface enhanced Raman spectroscopy, optical sensor and in biomedical application [15]. Colloidal AuNPs stabilized with phytochemicals in aqueous medium are required for many of its applications. In a bottom-up synthetic strategy, plant and fungi extract mediated solution phase synthesis of AuNPs involving reduction of Au(III) to Au(0) has gained profound significance in recent years because of the renewable and biocompatible nature of the plant and fungi extracts, eco-friendly aqueous medium and mild reaction condition [16–18]. Additional advantage of this method is that the extract itself acts as a stabilizer and no additional stabilizers are required. The extracts of *Aspergillus fischeri* [19], *Epicoccum nigrum* [20], bark of *Mimusops elengi* [21], leaf of *Chrysophyllum cainito* (Star apple) [22], bark of *Abroma augusta* Linn, [23] *Backhousia citriodora* [24], *Breynia rhamnoides* [25], *Piper betle* [26], pear extract [27] etc. have been utilized for the synthesis of AuNPs.

*Nerium oleander,* commonly known as Karabi, is an evergreen beautiful flowering shrub that belongs to the family Apocynaceae. The flowers of *Nerium oleander* grow in clusters in terminal branches, each 2.5–5 cm, funnel shaped with five lobes and white or pink colors. A number of plant secondary metabolites such as steroids, terpenoids, flavonoids, cardenolides, cardiac glycosides, long chain esters have been reported in the bark extract of *Nerium oleander* [28–31]. Tremendous biological effects such as heart tonic [32–35], diuretic [36], cytotoxic [37], antibacterial, anti-platelet aggregation [38–42], anti-inflammatory, hepatoprotective [43–46], antitumor, anti hyperlipidemic, anti-ulcer, anti-depressant action in central nervous system [47–53] have been reported. During our investigations on the utilization of triterpenoids (C30 s) as renewable functional nano entities [54–58] it occurred to us that the medicinally important bark extract of *Nerium oleander*, rich in polyphenolic compounds, can be utilized for the synthesis AuNPs from HAuCl_4_. In this paper we report the green synthesis of gold-conjugated nanoparticles by the reaction of aqueous chloroauric acid with an ethanol extract of the stem bark of the *Nerium oleander* under very mild reaction conditions without any additional stabilizing or capping agents. Synthesis of AuNPs carried out with increasing concentration of the bark extract showed that the smaller sized AuNPs form flower-like bigger-sized AuNPs composed of smaller sized AuNPs. The stabilized gold nanoparticles have been characterized by surface plasmon resonance (SPR) spectroscopy, high resolution transmission electron microscopy (HRTEM) and X-ray diffraction studies. Anticancer activity of the synthesized AuNPs studied against MCF-7 breast cancer cell lines indicated selective apoptosis of the cancer cells compared to normal cells. Catalytic activity of the synthesized AuNPs has also been demonstrated for model chemical transformations in aqueous medium at room temperature.

## Experimental

### Preparation of stem bark extract of *Nerium oleander*

Air dried stem bark of *Nerium oleander* (white flower variety) was finely powdered using a grinder. Finely powdered stem bark of *Nerium oleander* (7.5 g) was suspended in methanol (50 mL) and refluxed with magnetic stirring for 2 h, cooled at room temperature and then filtered via a sintered glass funnel. Volatiles of the filtrate were removed under reduced pressure to afford a sticky solid (1.00 g). The stem bark extract (0.100 g) was dissolved in distilled water (10 mL) and sonicated in an ultra sonicator bath for 10 min to get a semi-transparent solution (10 mg/mL).

### Synthesis of gold nanoparticles

Aliquots of Au(III) solution (0.2 mL, 11.6 mM each) were added drop wise to the stem bark extract solution of *Nerium oleander* contained in a vial (4 mL) and the final volume was made up to 4 mL to prepare a series of stabilized AuNPs where concentration of the bark extract varied from 200, 400, 600, 800, 1200–4000 mg/L, keeping the concentration of Au(III) fixed (0.58 mM). UV–visible spectroscopic measurement of the solutions were carried out after 7 h of mixing of HAuCl_4_ and the stem bark extract of *Nerium oleander*.

### Procedure of the catalytic reduction

To study the catalytic activity of the green synthesized colloidal AuNPs using the stem bark extract of *Nerium oleander*, two model reactions were carried out (a) the reduction of 3-nitrophenol to 3-aminophenol and (b) the reduction of 4-nitrophenol to 4-aminophenol, by sodium borohydride (16.38 mM) at room temperature in the presence of freshly synthesized colloidal AuNPs. Both the reactions were monitored by UV–visible spectroscopy.

### DPPH assay

A semi-transparent yellowish solution of the stem bark extract of *Nerium oleander* in ethanol was further diluted with ethanol to prepare a series of the extract with increasing concentration. Then ethanolic solution of DPPH (0.04 mL, 5.58 mM) was added to each solution of the extract and the volume was made up to 4 mL having final concentrations of the stem bark extract as 50, 100, 150 and 200 μg/mL. All the solutions were mixed thoroughly and then allowed to stand in the dark for 1 h at room temperature. The UV–visible spectrum of the colored solution was measured and the absorbance at 517 nm was noted. Reduction in absorption intensity of DPPH in the solutions containing the stem bark extract was observed when compared with a control solution of DPPH in ethanol at the same concentration. % scavenging was calculated using the following formula.

$$\% {\text{ DPPH radical scavenging activity}}\, = \,\left( {{\text{OD}}_{\text{Control}} - {\text{OD}}_{\text{Sample}} /{\text{OD}}_{\text{Control}} } \right)\, \times \, 100.$$

### Characterization

HRTEM images of AuNPs were recorded in JEOL JEM-2100 instrument. X-ray diffraction (XRD) patterns of the stabilized AuNPs were recorded Rigaku Miniflex II diffractometer with Cu-Kα radiation (λ = 1.54 Å). Mass spectra were recorded in Shimadzu GCMS QP 2100 Plus instrument. UV–visible spectrophotometry was carried out in Shimadzu 1601 spectrophotometer. For HRTEM analysis one drop of colloidal gold nanoparticles was placed over a carbon coated copper grid, allowed to dry in air and then under reduced pressure.

### Cell culture and maintenance

MCF-7 breast cancer cell line was obtained from Jadavpur University, Kolkata, India. The cell line was cultured in DMEM complete media with 10% FBS (fetal bovine serum). l-glutamine (2 mM), penicillin (100 U/mL), streptomycin (100 μg/mL) were required for the culture of the cell. Cultivated cells were incubated under 5% CO_2_ at 37 °C temperature in a CO_2_ incubator. Cells were grown in an exponential form until it reaches 1 × 10^6^ cells/mL growth.

### Selection of subjects for lymphocytes

Lymphocytes were separated from six different human samples belonging to the same geographical area and having the same environmental condition. The subjects were devoid of any kind of drug and anti-oxidant supplementation. Written consents were provided by these patients. Total process of lymphocytes separation was abided by Helsinki [59]. Ethical committee of Vidyasagar University had approved the process.

### Isolation of human lymphocytes

Blood samples (5 mL) were collected from six healthy persons by vein-puncture in a heparin coated vacutainers according to the method of Hudson and Hay [60]. Blood was diluted with PBS (Phosphate Saline buffer, 1:1 v/v) and centrifuged using Histopaque 1077 (Sigma) at 1500 rpm for 40 min for separation of the layers. Then the separation of lymphocytes were carried out following the previously described method.

### Intracellular reactive oxygen species generation

Intracellular ROS measurement was performed using H2DCFDA according to the procedure of Das et al. [59]. The drug (100 μg/mL) was treated with 2 × 10^5^ cells/mL for 24 h. After the treatment schedule, cells were washed with culture media followed by incubation with 1 μg/mL H2DCFDA for 30 min at 37 °C. Then the cells were washed three times with fresh culture media. DCF fluorescence was determined at 485 nm excitation and 520 nm emission using a Hitachi F-7000 Fluorescence Spectrophotometer. All the measurements were carried out in triplicate.

## Results and discussion

### Synthesis of AuNPs, UV–visible spectroscopy, HRTEM, DLS and XRD studies

**Fig. 1:**
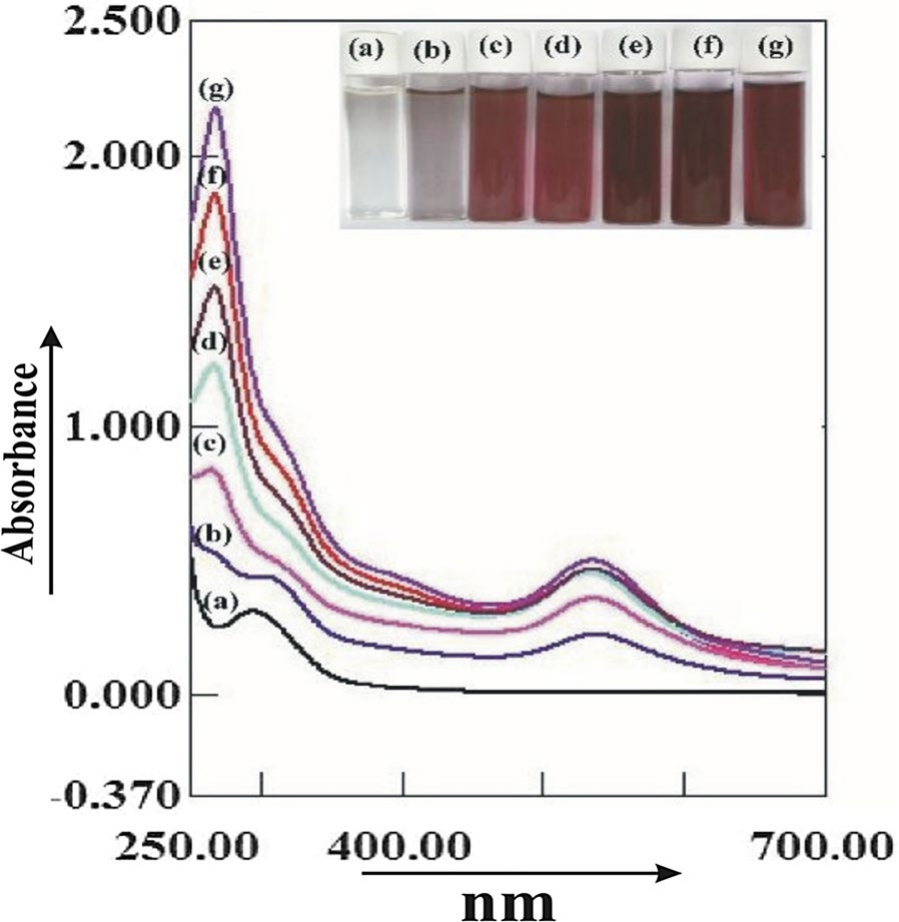
UV–Vis spectra of *a* HAuCl_4_ (0.42 mM), *b*–*g* AuNPs at 200, 400, 600, 800, 1000, 1200 mg/L concentrations of the stem bark of *Nerium oleander*. Inset: photograph of the vials containing the aforementioned mixtures (after 15 h of mixing)

Mass spectroscopy of an ethanolic extract of *Nerium oleander* bark in our laboratory indicated the presence of several polyphenolic compounds including flavonoids along with steroids and other plant secondary metabolites (Additional file 1: Figure S1). As the phenolic compounds are known to reduce Au(III) to Au(0), we treated a series of the solution of the stem bark extract (with increasing concentration from 200 to 1200 mg/L) contained in vials (capacity 4 mL) with an aqueous solution of chloroauric acid (with the final concentration of Au(III) of 0.58 mM). Chloroauric acid has an absorption peak at 290 nm in the UV–visible spectrum due to charge transfer interactions between the metal and the chloro ligands. The formation of gold nanoparticles from *Nerium oleander* stem bark extract was evident from the UV–visible spectroscopy studies (Fig. 1). The surface plasmon resonance (SPR) band in the 534–553 nm region were formed upon addition of HAuCl_4_ solution to the stem bark extract of *Nerium oleander* indicating the formation of AuNPs. Increasing the concentration of the stem bark extract from 200 to 1200 mg/L resulted in blue shift of the SPR band. The blue shift of the SPR band is indicative of the formation of smaller sized AuNPs with increasing concentration of the bark extract.

**Fig. 2:**
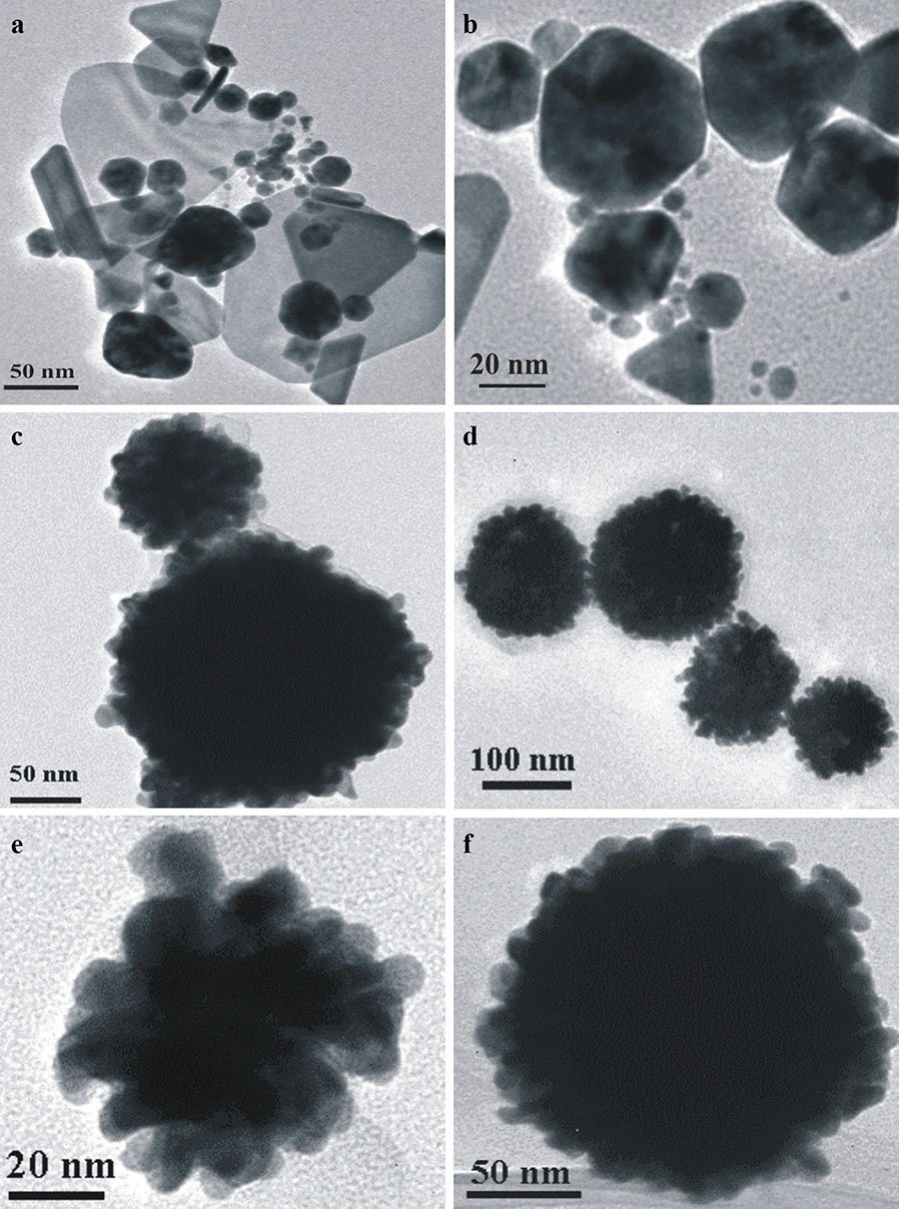
HRTEM images of stabilized AuNPs at various concentration of the stem bark extract: **a**, **b** concentration 800 mg/L; **c**–**f** concentration 4000 mg/L

The morphology of stabilized AuNPs synthesized using the stem bark extract of *Nerium oleander* was investigated by high resolution transmission electron microscopy (HRTEM) analysis (Fig. 2). HRTEM images of dried samples prepared from three colloidal AuNPs samples containing 800, 1200 and 4000 mg/L concentration of the stem bark extract of *Nerium oleander* were recorded. Mostly spherical shaped AuNPs of 20–40 nm size were observed by HRTEM along with hexagonal, triangular and rod like particles at a lower concentration of the bark extract (800 mg/L). Flower-like of AuNPs having 100–300 nm diameters composed of smaller sized AuNPs of 5–10 nm were observed at higher concentration of the bark extract (4000 mg/L). DLS studies carried out with AuNPs synthesized using 400 mg/L of the bark extract indicated that the size of green synthesized particles were 10–100 nm (Additional file 1: Figure S2). The synthesized AuNPs were highly stable when stored at room temperature and no aggregation of the colloidal AuNPs was observed for several months. This high stability can be explained by the very high negative zeta potential value of − 16.3 mV (Additional file 1: Figure S3).

**Fig. 3:**
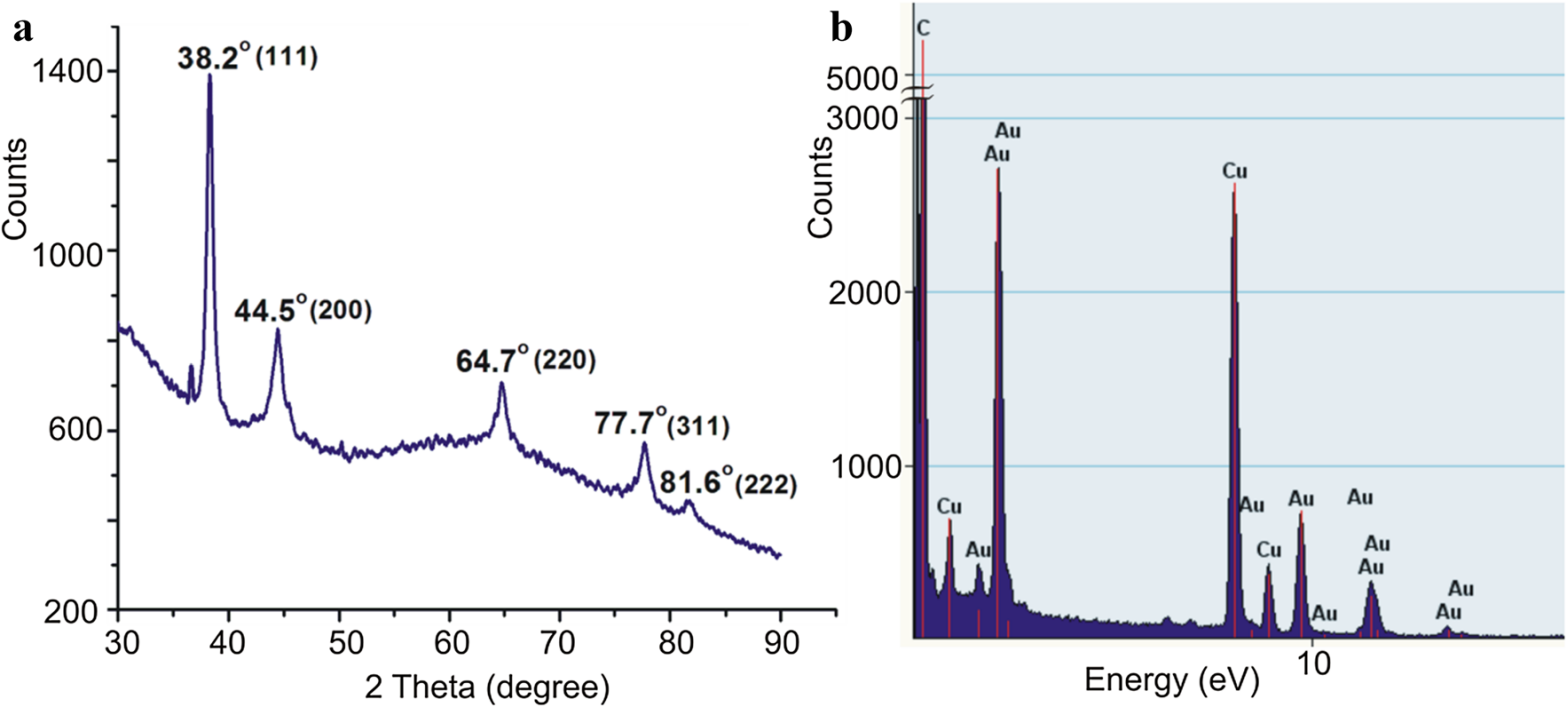
**a** XRD, **b** EDX of stabilized AuNPs synthesized with the bark extract of *Nerium oleander*

To investigate the nature of the stabilized AuNPs, a dried sample of AuNPs coated over a glass plate was analyzed by X-ray diffraction studies (Fig. 3). The reflections of the planes (111), (200), (220), (311) and (222) observed at 2θ = 38.2°, 44.5°, 64.7°, 77.7° and 81.6° respectively supported reduction of Au(III) to Au(0) by the phytochemicals present in the stem bark extract of *Nerium oleander* and also confirms the crystallinity of gold atoms. The face centered cubic (fcc) structure of the crystalline AuNPS were confirmed by comparison with the standard data given by JCPDS file no. 04-0784. The intensity of the (111) plane comparatively other plane indicated the predominant orientation of the (111) plane. EDX analysis of the stabilized AuNPs also indicated the presence of gold nanoparticles (Fig. 3b) stabilized by plant phytochemicals.

### Mechanism of the formation of stabilized AuNPs

**Fig. 4:**
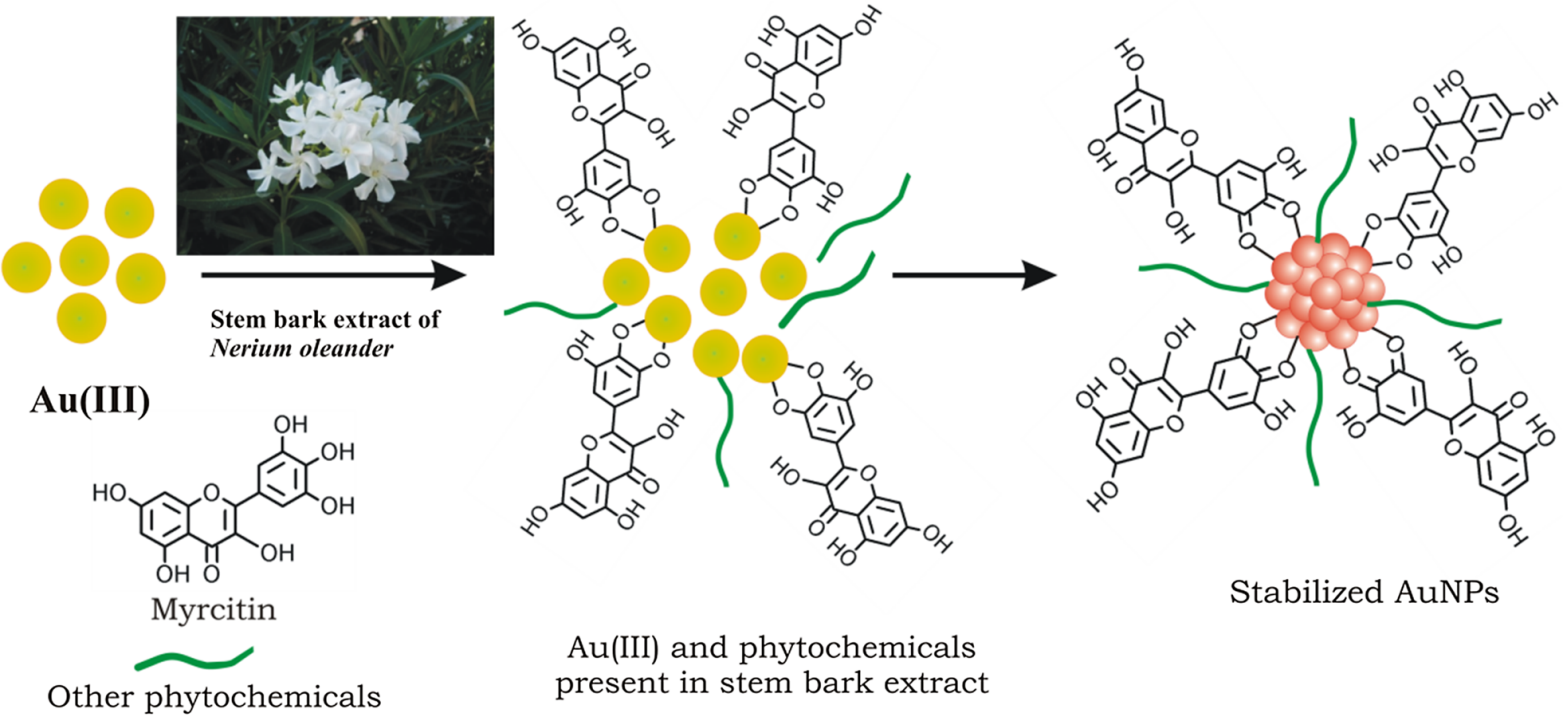
Mechanism of the formation of stabilized AuNPs by polyphenolic compound and phytochemicals present in the stem bark extract of *Nerium oleander* by taking myricetin as a representative of polyphenols

The stem bark extract of *Nerium oleander* is rich in different types of plant secondary metabolites such as polyphenols including flavonoids, steroids, etc. Mass spectral analysis of the stem bark extract carried out by us supported the presence of the several polyphenolic compounds and terpenoids such as myricetin (M^+^ 318), quercetin (M^+^ 302), betulin (M^+^ 442), betulinic acid (M^+^ 456) or their analogues. Taking the *o*-dihydroxyphenolic compound myricetin as a representative, a schematic representation of a plausible mechanism for the synthesis of stabilized gold nanoparticles is shown in Fig. 4. The *o*-dihydroxy group of the polyphenolic compounds can coordinate with the Au(III) ion and form a five membered chelate ring. The chelated Au(III) ion was reduced to Au(0) with concomitant oxidation of the *o*-dihydroxyl groups to quinones because of very high oxidation–reduction potential of Au(III). Au(0) atoms can collide with neighboring Au(0) atoms forming AuNPs which are then stabilized by the polyphenolic compounds as well as quinones.

### Application of stabilized AuNPs

To demonstrate the usefulness of the stabilized AuNPs, in vitro anticancer activity and catalytic activity were studied which are discussed in the following sections.

#### Application of stabilized AuNPs as an anti-cancer drug

To find out whether AuNPs can act as a drug, dose dependent cytotoxicity assay of the freshly prepared AuNPs (0.5 mL, 1000 mg/L, synthesised with HAuCl_4_ (0.58 mM) and the stem bark extract (400 mg/L) was carried out on MCF-7 cell line. Non-radioactive colorimetric assay technique using tetrazolium salt, 3-[4,5-dimethylthiazole-2-yl]-2,5-diphenyl tetrazolium bromide (MTT) was used for the cell viability studies.

The % of cell viablility was calculated by using the following equation [59].

$$\% {\text{ of cell viability }} = \, \left[ {{\text{OD}}_{\text{sample}} {-}{\text{ OD}}_{\text{control}} } \right] \times 100/{\text{OD}}_{{{\text{control}}.}}$$

**Fig. 5:**
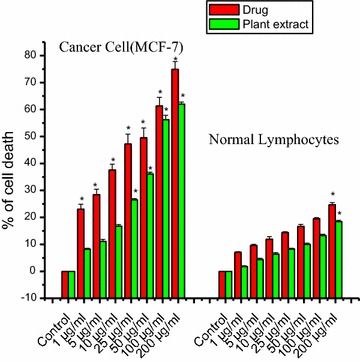
In vitro cell viability assay of drug (synthesized AuNPs) and plant extract treated on normal PBL and MCF-7 cell-lines. Cells were treated with the drug and the plant extract for 48 h at 37 °C. Cell viability was measured by the MTT method as described in materials and methods. Values are expressed as mean ± SEM of experiments; superscripts indicate significant differences (p < 0.05) compared with the control group

From the graph which is obtained from MTT assay, we calculated the % of cell death in case of both the cancer cell line as well as in normal lymphocytes after the treatment of the drug (AuNPs) and the plant extract. Both the drug and plant extract showed potent anti-cancer activity but the AuNPs are more efficient compared to the bark extracts. The killing ability of the drug (AuNPs) against breast cancer cell line was 23.01, 28.43, 37.59, 47.22, 49.49, 61.30, 74.87% which were significantly higher compared to that of the plant extract (8.07, 11.39, 17.65, 26.72, 33.62, 54.31 and 61.22% respectively) at 1, 5, 10, 25, 50, 100, 200 μg/mL dose (Fig. 5).

The half inhibitory concentration (IC_50_) values for the AuNPs drug and the plant extract against the cancer cell MCF-7 were 74.04 and 130.87 μg/mL respectively (Additional file 1: Figure S4). Multiple linear regressions were used for comparison of data through statistica version 5.0 (Stat soft, India) software package. The anticancer activity of the plant extract, though less effective compared to the AuNPs drug (as obvious from the above mentioned IC_50_ values), might be due to the antioxidants present in the extract (as evident from the DPPH assay discussed in Sect. 3.4) [61].

The cytotoxicity assay was carried out against normal lymphocytes. The % of lymphocyte killing is more in case of the drug compared to the plant extract. 100 μg/mL dose of the drug can be used as a biologically safe dose because at this particular dose it killed the cancer cell massively and the cytotoxicity is minimum. From the point of cytotoxicity, the plant extract is better but the anti-cancer activity was insignificant compared to the drug. AuNPs are more efficient to kill the cancer cells compared to the plant extract.

#### Intracellular reactive oxygen species (ROS) measurement

**Fig. 6:**
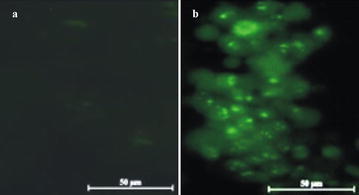
ROS production by the drug (synthesized AuNPs) **a** is the control MCF-7 cell line **b** is the drug treated MCF-7 cell line

ROS are unpaired valance shell electrons. They are able to damage the cells and ultimately cell death [62]. The physiological system produce very lower amount of ROS due to various metabolic activity. Several anti-oxidant enzymes help to neutralize them. ROS are generally produced from mitochondrial respiratory chain. Super oxides are generated by nicotinamide adenine dineucloetide phosphate (NADPH) oxidase reaction [63]. In the present study, we have treated the MCF-7 cells with the synthesized AuNPs at 100 µg/mL dose to find out the efficacy of ROS production by the drug to kill the cancer cells. Microscopic image reveals the ROS generation in the cancer cells (Fig. 6). Simultaneously the drug does not remarkably effect the normal lymphocytes (Additional file 1: Figure S5). So, this drug can be very effective for further cancer treatment.

### Determination of antioxidant activity of the bark extract by DPPH assay

**Fig. 7:**
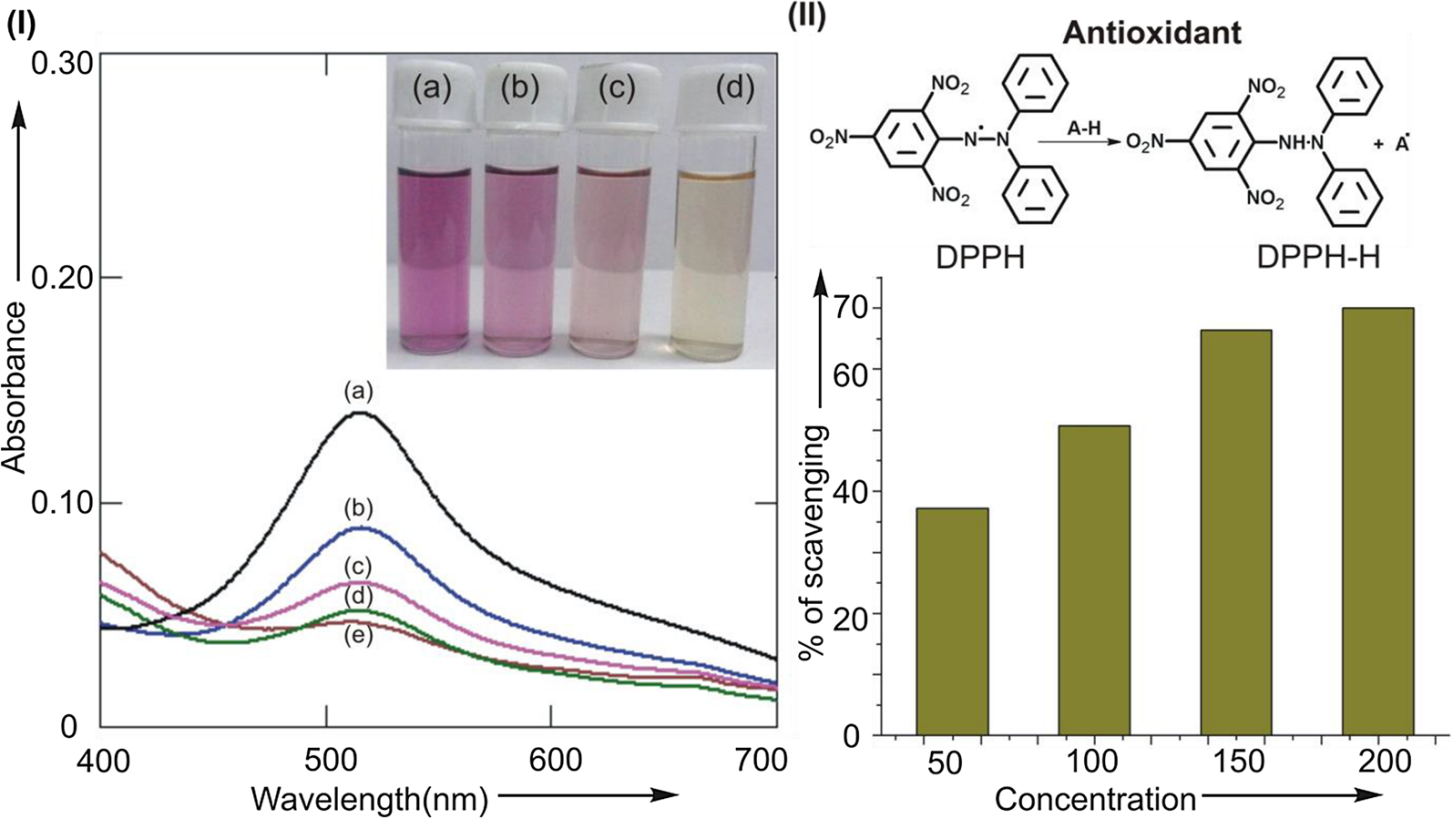
Antioxidant activity studies of the stem bark extract of *Nerium oleander*: (**I**) UV–visible spectra of (a) DPPH, (b–e) DPPH + with ethanol extract of stem bark 50, 100, 150 and 200 μg/mL. Inset: photographs of the vials containing the solutions of (a) DPPH, (b–d) DPPH + with ethanol extract of stem bark 50, 100 and 150 μg/mL. **II** Reaction scheme showing quenching of DPPH radical by the antioxidant (A–H); (**III**) plot of % DPPH radical scavenging by the ethanol extract stem bark

The 2,2-diphenylpicrylhydrazyl (DPPH) is widely used in plant biochemistry to evaluate the properties of plant constituents for scavenging of free radicals. The free radical scavenging activity of the stem bark extract of *Nerium oleander* was tested against the ethanol extracts of DPPH following the procedure described by us previously [23]. 2,2-diphenylpicrylhydrazyl radical reacts with the antioxidant (A–H) and convert it to 1-1-diphenyl-2-picryl hydrazine and a change in color was observed (Fig. 7). The degree of decolorization indicates the scavenging potential of the plant extract. % radical scavenging activity was calculated to be 70 when concentration of the bark extract of *Nerium oleander* is 200 μg/mL. This radical scavenging property of the stem bark extract might explain its anticancer activity towards MCF-7 cell lines as discussed in Sect. 3.3.1.

### Catalytic activity

AuNPs with very high surface to volume ratio of have recently been utilized as a catalyst for various kinds of chemical transformations. To test whether the stem bark extract of *Nerium oleander* derived colloidal AuNPs can be utilized as a catalyst for photocatalytic reduction of toxic pollutants, we chose the sodium borohydride reduction of 3-nitrophenol to 3-aminophenol and 4-nitrophenol to 4-aminophenol as model reactions.

#### Reduction of 3-nitrophenol to 3-aminophenol by stabilized AuNPs

**Fig. 8:**
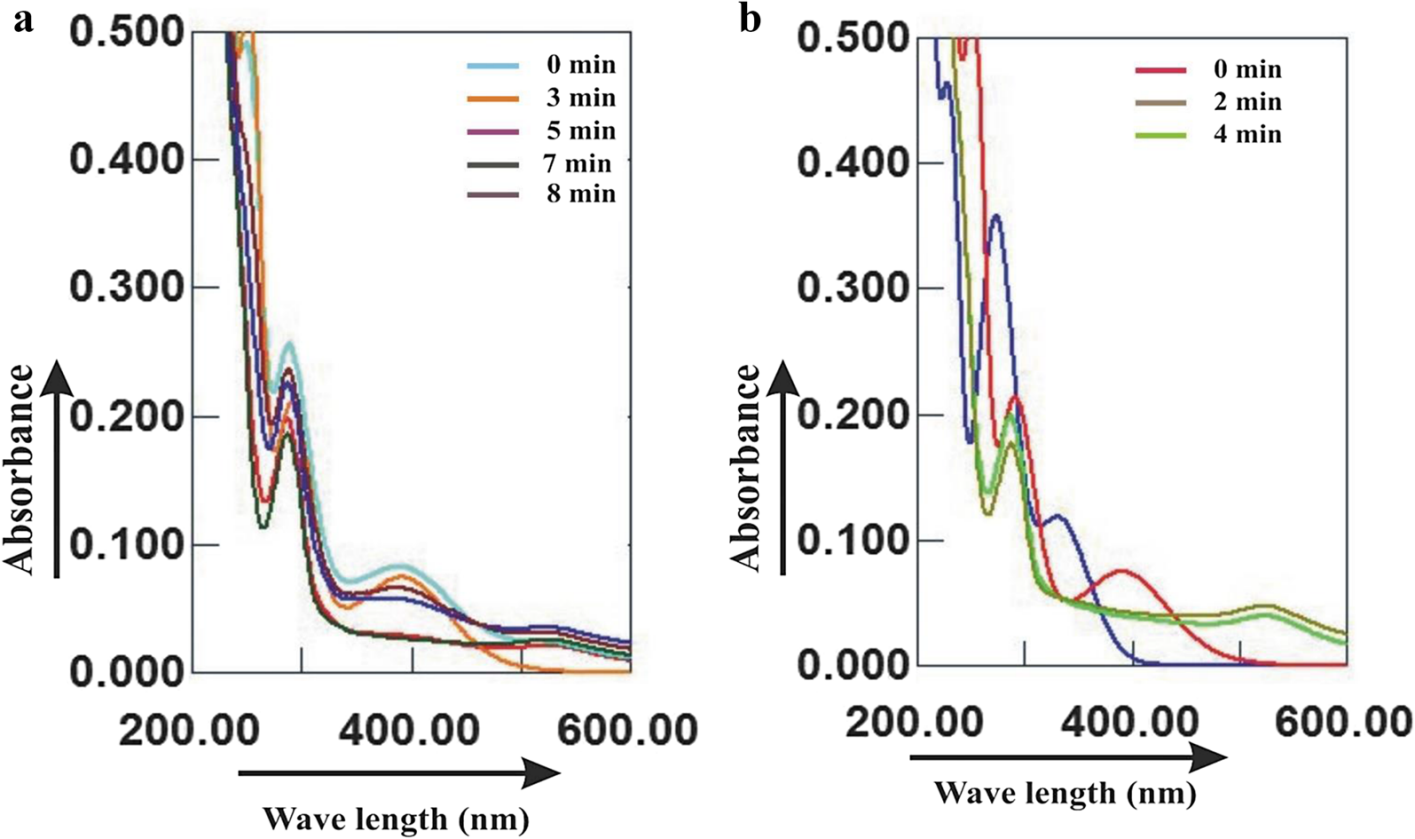
UV–visible spectra at different time interval during the catalytic reduction of 3-nitrophenol to 3-aminophenol using **a** 0.05 mL and **b** 0.1 mL of stabilized AuNPs

On treatment of an aqueous solution of 3-nitrophenol (0.05 mM) with sodium borohydride (16.39 mM) at room temperature, the absorption band at 331 nm shifted to 392 nm due to the formation of 3-aminophenolate anion. But due to a large kinetic barrier for the reduction reaction, no reduction of the nitro to amino took place even though borohydride reduction of nitro group is a thermodynamically favourable reaction. On addition of 0.05 mL of stabilized AuNPs (synthesized with chloroauric acid (0.58 mM) and the bark extract (400 mg/L) to the reaction mixture, the peak at 392 nm peak slowly reduced and completely disappeared in 8 min indicating the formation of 3-aminophenol (Fig. 8). This demonstrated the catalytic activity of the stabilized AuNPs. For this reduction reaction, the rate constant was calculated as 1.43 × 10^−3^ s^−1^ that is consistent with the values obtained by us and others reported previously [16, 21]. Similarly on addition of 0.1 mL of stabilized AuNPs to the reaction mixture, the peak intensity at 392 nm decreased rapidly and completely disappeared in 2 min. The rate constant for this reduction reaction could not be calculated due to very rapid transformation.

#### Reduction of 4-nitrophenol to 4-aminophenol by stabilized AuNPs

**Fig. 9:**
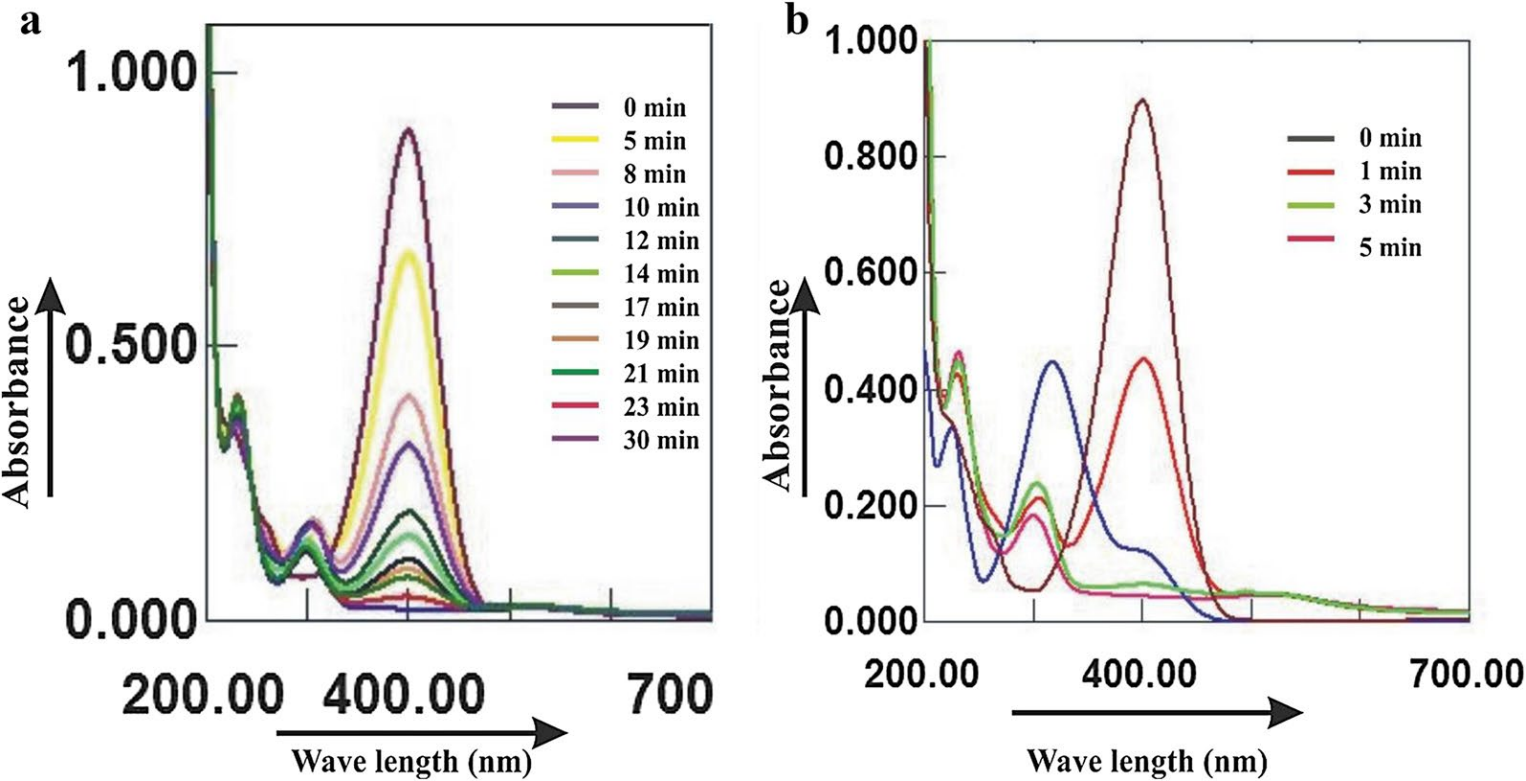
UV–visible spectra at different time interval during the catalytic reduction of 4-nitro phenol to 4-amino phenol using **a** 0.05 mL and **b** 0.1 mL of stabilized AuNPs

On treatment of an aqueous solution of 4-nitrophenol (0.05 mM) with sodium borohydride (16.39 mM) at room temperature, the absorption band of 4-nitrophenol at 318.5 nm shifted to 401 nm due to the formation of 4-nitrophenolate anion. But no reduction of the nitro group took place even on standing the reaction mixture at room temperature for several days. On addition of 0.05 mL of stabilized AuNPs (synthesized with chloroauric acid (0.58 mM) and the bark extract (400 mg/L) to the reaction mixture, the intensity of the peak at 401 nm for the 4-nitrophenolate ion decreased gradually and completely disappeared in 30 min with concomitant appearance of a new peak at 401 nm indicating the formation of 4-aminophenolate (Fig. 9). The rate constant value from UV–Vis data was calculated to be 2.198 × 10^−3^ s^−1^. Similarly on addition of 0.1 mL of stabilized AuNPs to the reaction mixture, the peak for 4-nitrophenolate ion completely disappeared in 5 min with concomitant appearance of a new peak for 4-aminophenolate ion. The rate constant value for this reduction reaction was calculated to be 1.23 × 10^−2^ s^−1^ that is in consistent with the values reported by us and others previously [23, 25].

## Conclusion

The phytochemicals present in the stem bark extract have been utilized for the synthesis of gold nanoparticles at room temperature under very mild conditions without any additional stabilizing agents. The antioxidant activity of the stem bark extract has been studied against the long lived 2,2-diphenylpicrylhydrazyl (DPPH) radical at room temperature. According to our knowledge, this is the first report of the study of antioxidant property of the stem bark extract of *Nerium oleander* and its utilization in the green synthesis of gold nanoparticles. A mechanism for the synthesis of the gold nanoparticles has also been proposed. The present study also demonstrated the in vitro anticancer activity of the stabilized AuNPs on MCF-7 cell lines significantly killing the cancer cells at 74 μg/mL. The normal lymphocytes were found to be non-toxic at this dose. The catalytic activities of the stabilized AuNPs have also been demonstrated for borohydride reduction of 3- and 4-nitrophenols.

## Additional file

**Additional file 1.** Mass spectra, DLS data, plot of % cell death vs dose of drug, IC_50_ value, effects of drug on ROS induction in Lymphocytes and MCF-7 cell lines.
