# Supplementary material for: Green synthesis of Nerium oleander-conjugated gold nanoparticles and study of its in vitro anticancer activity on MCF-7 cell lines and catalytic activity
Source: Nano Converg. 2018 Apr 19;5:10. doi: 10.1186/s40580-018-0142-5 (PMC5906526; doi:10.1186/s40580-018-0142-5)
Supplement: Supplementary file 1 — Additional file 1. Mass spectra, DLS data, plot of % cell death vs dose of drug, IC50 value, effects of drug on ROS induction in Lymphocytes and MCF-7 cell lines. [file 40580_2018_142_MOESM1_ESM.doc]

Additional file

**Green synthesis of gold-conjugated *Nerium oleander* nanoparticles and study of its in-vitro anticancer activity on MCF-7 cell lines and catalytic activity**

**Abir Chandan Barai,^a^ Koushik Paul,^a^ Aditi Roy,^b^ Subhankar Manna,^b^ SomenathRoy,*^,b^ Braja Gopal Bag^,*,a^ Chiradeep Mukhopadhyay ^a^**

**^a^ Department of Chemistry and Chemical Technology,**

**^b^ Department of Human Physiology with Community Health,**

**^a,b^ Vidyasagar University, Midnapore 721102, West Bengal, INDIA**

**Email:** [***braja@mail.vidyasagar.ac.in***](mailto:braja@mail.vidyasagar.ac.in)

1. **Materials:**

Chloroauric acid (HAuCl4) was purchased from SRL (Sisco Research Laboratory) and used without further purification. The stem bark of *Nerium oleander*(white flower variety commonly known as swetkarabi) was collected from the garden of Vidyasagar University, West Bengal, India and identified at the Department of Botany and Forestry, Vidyasagar University. Sodium borohydride and 4-nitrophenol were purchased from Merck and DPPH was purchased from sigma and aldrich. Double distilled water was used for the synthesis of AuNPs and studies with MCF-7 breast cancer cell line.

**2. Preparation of Au (III) Solution:**


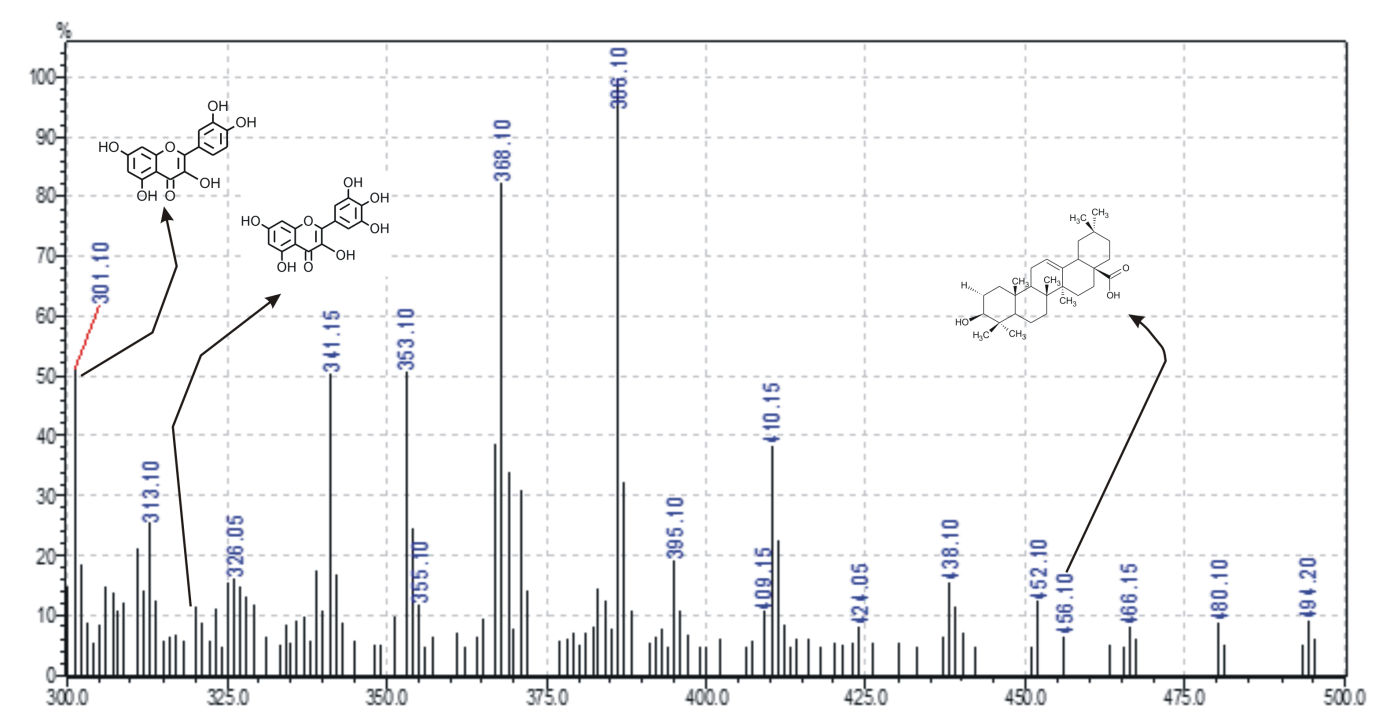
HAuCl4 (39.3 mg) was dissolved in distilled water (10 mL) to obtain a 11.6 mM Au(III) stock solution.

Figure S1: Mass spectrum of the bark extract of *Nerium oleander* (with white flower)


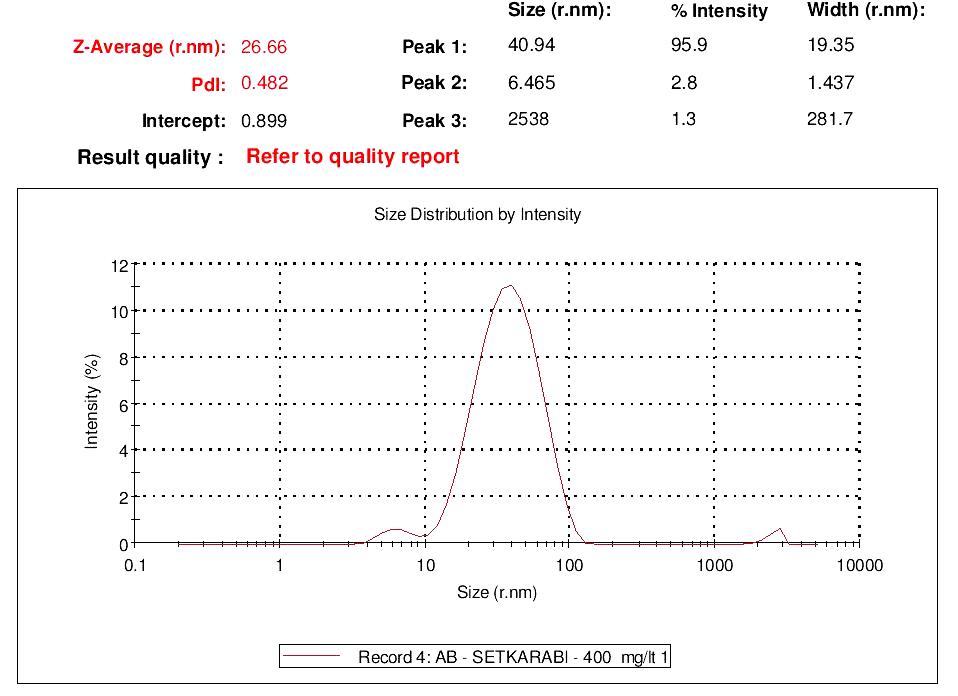


Figure S2: Avarage size of AuNPs synthesized with the stem bark extract of *Nerium oleander* (with white flower).


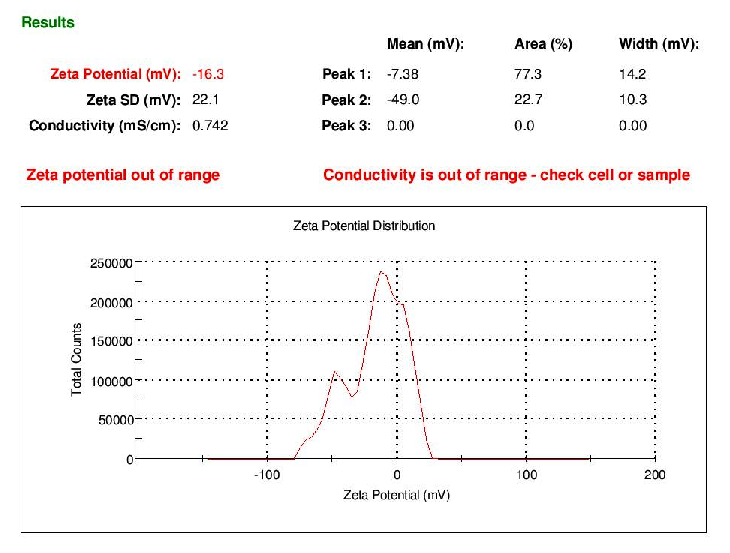


Figure S3: Zeta potential value from DLS studies of AuNPs synthesized with the stem bark extract of *Nerium oleander* (with white flower).


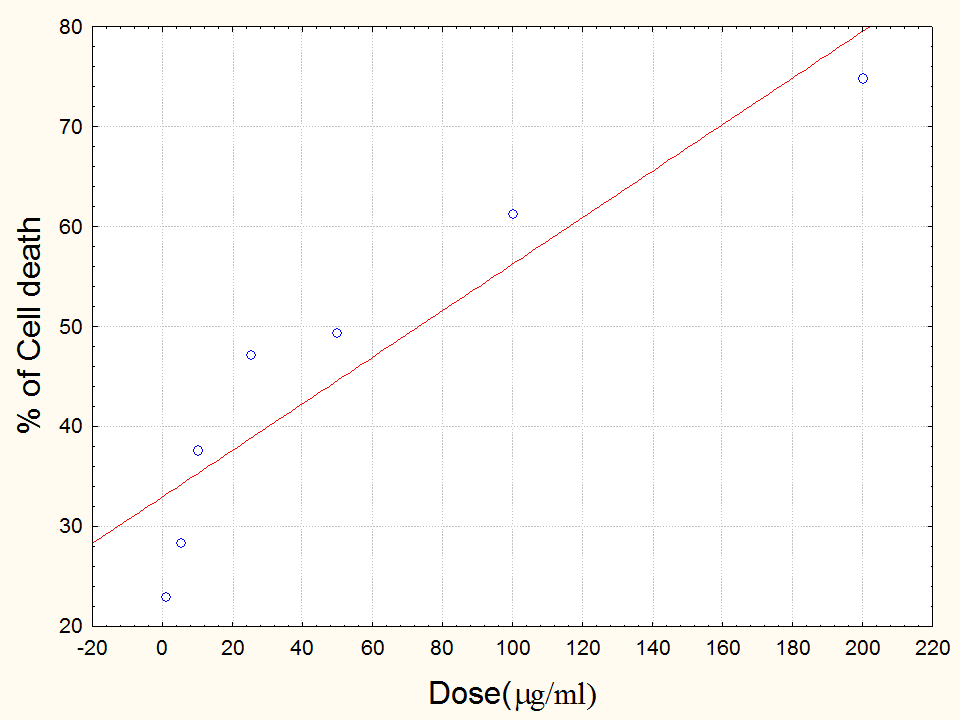


Figure S4: The IC 50 value of the drug AuNPs is = 74.04μg/mL


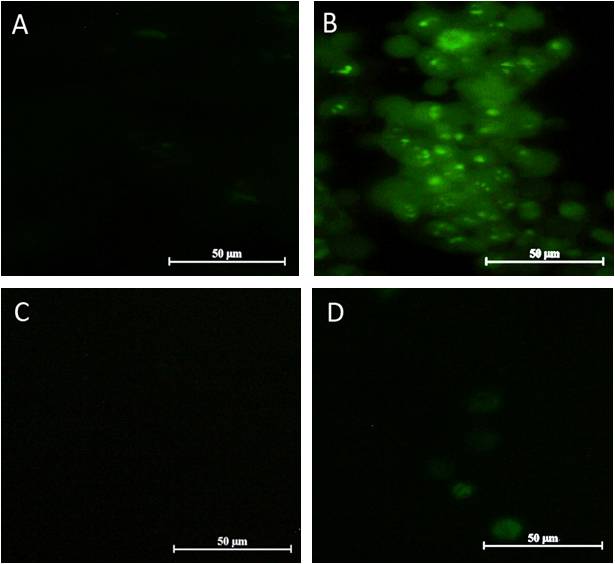


Figure S5: Effects of Drug on ROS induction in Lymphocytes and MCF-7 cell lines. (A) is the control MCF-7 cells (B) is the drug treated MCF-7 cells (C) is the normal Lymphocytes and (D) is the drug treated Lymphocytes
